# Perisciatic Nerve Dexmedetomidine Alleviates Spinal Oxidative Stress and Improves Peripheral Mitochondrial Dynamic Equilibrium in a Neuropathic Pain Mouse Model in an AMPK-Dependent Manner

**DOI:** 10.1155/2022/6889676

**Published:** 2022-06-20

**Authors:** Yang Mu, Yang Mei, Yaohua Chen, Yuping Li, Dan Zhu, Jian Cui, Lehua Yu

**Affiliations:** ^1^Department of Rehabilitation Medicine, The Second Affiliated Hospital of Chongqing Medical University, Chongqing 400010, China; ^2^Department of Rehabilitation Medicine, Chongqing University Fuling Hospital, Chongqing 408099, China; ^3^Department of Pain Medicine, Chongqing Traditional Chinese Medicine Hospital, Chongqing 400021, China; ^4^Department of Pain Medicine, The First Affiliated Hospital, Army Medical University, Chongqing 400038, China

## Abstract

Neuropathic pain (NPP) is a debilitating clinical condition that presently has few effective treatments. NPP is caused by uncontrolled central oxidative stress and inflammation. Preliminary studies indicate that dexmedetomidine (DEX), an agonist of the alpha-2 adrenergic receptor, is beneficial for treating NPP. In this paper, the effects of administering DEX around injured nerves in a chronic constriction injury- (CCI-) induced neuropathic pain mouse model are investigated. According to the results, the perineural DEX significantly reversed the decline in the mechanical threshold and thermal latency in CCI mice (*p* < 0.001). In the peripherally affected ischiadic nerve, the perineuronal DEX upregulated the expressions of pAMPK, OPA1, and SNPH but not Drp1 or KIF5B. The aforementioned effects of administering DEX can be partially reversed by compound C, a selective and reversible inhibitor of AMP-activated protein kinase (AMPK). Furthermore, it was found that perineural DEX significantly inhibited the CCI-induced upregulation of the immediate early gene c-Fos, overexpression of the inflammatory factors tumor necrosis factor-alpha (TNF-*α*) and interleukin-6 (IL-6), attenuation of the NADH dehydrogenase complexes I, II, III, and IV, and the repression of ATP, SOD, and GSH in the dorsal horn of the spinal cord (DHSC) (*p* < 0.01). These findings indicate that perineuronal DEX protected the injured ischiadic nerves and attenuated neuropathic pain via AMPK activation to improve energy supply in the peripheral injured nerves, alleviate the inflammatory factor release, and inhibit oxidative stress in the DHSC.

## 1. Introduction

Neuropathic pain (NPP) is one of the most common refractory conditions of chronic nature. Although multiple approaches have been explored, its treatment remains a challenge to internists. Although the impact of the mitochondrial disorder on the induction and maintenance of spinal oxidative stress and inflammatory cytokine accumulation has previously been reported, the underlying mechanism remains unclear [1, 2]. AMP-activated protein kinase (AMPK), a strongly conserved and highly expressed eukaryotic protein serine/threonine kinase, is capable of sensing changes in body energy and has a crucial role in modulating mitochondrial function [3], combating oxidative stress and inflammation [4]. DEX, an *α*2-adrenoceptor agonist, has calming, anxiolytic, and antalgic properties with minimal respiratory inhibition [5]. He et al. reported that in a murine model of spinal cord impairment, DEX can exert anti-inflammatory effects through AMPK pathway initiation [6]. Moreover, DEX reportedly reduced the impairment of brain tissues by facilitating cerebral AMPK expression and lowering inflammatory cytokine secretions [7]. This study is aimed at observing whether DEX has therapeutic effects on the CCI-induced NPP following perineural injection and at further exploring its underlying mechanism.

## 2. Methods and Materials

### 2.1. Animals

Male C57BL/6 mice weighing 20–25 g from the Laboratory Animal Institute of Army Medical University in China were treated according to the guidelines of the National Institutes of Health, which emphasizes pain minimization and quantitative limitation of experimental animals. The mice were housed at a constant temperature of 25°C under a 12 h diurnal-nocturnal cycle and were fed and watered ad libitum. This experiment was approved by the Laboratory Animal Welfare and Ethics Committee of the Army Military Medical University (AMUWEC20210716).

### 2.2. Experimental Groups

#### 2.2.1. Part 1

Based on the ligation and therapeutic regimes, the mice were randomly divided into 6 groups using a computer random number generation program (*n* = 12), namely, the CCI+saline, the CCI, the CCI+DEX (12.5 *μ*g/kg), the CCI+DEX (25 *μ*g/kg), the CCI+DEX (50 *μ*g/kg), and the negative control groups. The DEX was injected locally around the sciatic nerve daily after the CCI operation, and the pain threshold was tested on days 1, 3, 7, and 14.

#### 2.2.2. Part 2

For subsequent studies, a 14-day time point and a DEX dose of 25 *μ*g/kg body weight were selected based on part 1 results. The mice were randomly grouped into four (*n* = 12): the CCI, the CCI+DEX, the CCI+DEX+CC, and the negative control groups.

### 2.3. CCI Model and Local Injection at the Sciatic Nerve

CCI modeling was accomplished as per the procedure outlined by Bennett and Xie [8]. The mice were anesthetized by isoflurane inhalation, and the left sciatic nerve of each mouse was exposed. The braided 4.0 silk thread (Ethicon, Belgium) in the nerve periphery was utilized to make 2 loose ligatures that were 1 mm apart, followed by a layer-wise closure of the incision. The perineural injection was administered as per the procedure described by Leszczynska and Kau [9]. Forceps were utilized to hold the right posterior limb at the knee of each mouse after each mouse was placed with its head down into a plastic tube with a height of 4.5 and a diameter of 1.2. A 27-gauge (1 inch) needle with a tuberculin syringe was utilized for injecting the experimental compound into the popliteal space in the sciatic nerve region (of the posterior knee surface) directly at the rear tip of the forceps. A fixed volume of 0.05 mL was injected.

### 2.4. Determination of Mechanical Threshold and Heat Latency

The mice were placed on a test table made of metal mesh, and after 30 min of adaptation, the von Frey cilia were vertically stimulated at the bottom and middle of the mice in order from low to high (0.16, 0.4, 0.6, 1.0, and 1.4) to allow persistent bending of the cilia for at least 2 s in the stimulation process. On every mouse, five cilia stimulations were implemented at identical intensities, during which the behavioral performance of the mouse was recorded. The pain-associated behaviors like leg lifting or swinging, foot contraction, and licking were documented as positive reactions. The mechanical threshold was defined as the lowest stimulus intensity of the 3 positive reactions, which is the 50% paw-withdrawal mechanical threshold (PWMT).

The mice were arranged on an experimental bench for radiative heating, and their activities were restricted by Plexiglas boxes. After 30 min of adaptation, the skin of each mouse was stimulated in turn, and the threshold of the pain-related behavioral response of the mice was recorded. The incubation period of leg retraction was recorded from exposure to leg retraction. Each hind paw was tested every 5 min for a total of five times. The computed average determines the threshold of thermal sensitivity of the mouse, which is the paw withdrawal thermal latency (PWTL).

### 2.5. Assessment of the Inflammatory Cytokines

After homogenizing the L4–L6 spinal dorsal horns (SDHs) with the tissue protein extraction (T-PER) reagent, the supernatant was collected after 15 min of centrifugation at 4°C and 4000 rpm. The mouse-specific ELISA kits (eBioscience, USA) were utilized to assess interleukin-6 (IL-6) and tumor necrosis factor-alpha (TNF-*α*), as per the guidelines of the manufacturer for sealing, adding samples, incubating, and developing color. The optical density (OD) was measured at 450 nm using a microplate reader (VarioSkan Flash, Thermo Fisher Scientific, USA). The results are presented as pg/mg of protein following the protein level normalization based on the standard curve [10].

### 2.6. Mitochondrial Functionality Assays

The mitochondrial fractions were separated from the sciatic nerves as per the protocol of the manufacturer with the aid of the Mitosol Kit (Sigma, USA). The immunocapture assay kits (Abcam, UK) were utilized to assess the enzymatic activity for complexes I and II in the mitochondrial isolates separately at 450 and 600 nm, as per the manufacturer's guidelines. The results are presented as mOD/min/mg of protein after normalizing the protein level. To determine the cytochrome c reducing activity, a slightly revised version of the procedure of Kramer et al. [11] was adopted using a 96-well format. The ability of complex II+III to reduce cytochrome c was measured by calculating the increase in its 550 nm OD, which is expressed as mOD/min/mg of protein after normalizing the protein level. The activity assay of complex IV was accomplished at 550 nm using a cytochrome c oxidase kit from Sigma–Aldrich (USA) as per the instructions of the manufacturer.

The aerobic adenosine triphosphate (ATP) levels in all L4-L6 spinal cord segments were calculated using an ATP Assay Kit (Beyotime, China) as per the manufacturer guidelines. The superoxide dismutase (SOD) activity at 450 nm, the malondialdehyde (MDA) levels at 532 nm, and the glutathione (GSH) content at 420 nm in the sciatic nerves were measured using the commercial kits from the Jiancheng Bioengineering Institute (China) as per the guidelines.

### 2.7. Immunohistochemical Analysis

The sciatic nerve and the DHSC sections of thickness 5 *μ*m were immobilized with neutral-buffered formalin (10%) before being subjected to immunohistochemical (IHC) analysis as instructed by the PolyExcel HRP/DAB Detection System (PathnSitu Biotechnologies, India). After rehydration, primary antibodies against pAMPK, SNPH, KIF5B, OPA1, and Drp1 (1 : 200 diluents; Cell Signaling Technology, USA) were utilized to incubate the coronal sections of the sciatic nerves, while a 1 : 500 diluent of anti-c-Fos primary antibody from the same manufacturer was used for the incubation of the DHSC section. To visualize the cellular nuclei, tissue sections were counterstained with mounting media containing fluorescent DAPI (Vector Laboratories), followed by washing and hematoxylin counterstaining. Finally, for the section observation, an optical OPTIKA microscope (Model no. B-293) was utilized.

### 2.8. Western Blot Analysis

The sciatic nerve tissues were homogenized using T PER (Thermo Scientific, USA) involving a protease–phosphatase inhibitor cocktail (1%; Sigma, USA). Then, an identical amount of protein was loaded and isolated via SDS-PAGE gel and subsequently shifted onto a polyvinyl difluoride (PVDF) membrane. After a 1 h blockage with bovine serum albumin buffer (3%), the membrane was incubated overnight using the primary antibodies against pAMPK, SNPH, KIF5B, OPA1, Drp1, and *β*-actin (1 : 1000 dilution, Cell Signaling Technology, Beverly, MA, USA). The washed membranes were incubated with a 1 : 5000 diluent of HRP-labeled secondary antibody and visualized in chemiluminescent mode. The ImageJ 1.36 program (MD, USA) was utilized for the densitometric quantification of relative band densities. Then, the expression of *β*-actin was determined to evaluate equal protein loading.

### 2.9. Data Analysis

The data were expressed as means ± standard errors of the means (SEMs). The univariate analysis of variance (ANOVA) was performed on the end-point data for statistical processing, while bivariate ANOVA was performed to process the behavioral outcome data with the aid of Prism ver. 5.0 (GraphPad Software, USA). The Bonferroni multicomparison test was employed during the follow-up assessment. The differences were regarded as significant when the *p* values were less than 0.05.

## 3. Results

### 3.1. DEX Attenuated the Mechanical Threshold and Heat Latency in CCI Mice

**Figure 1:**
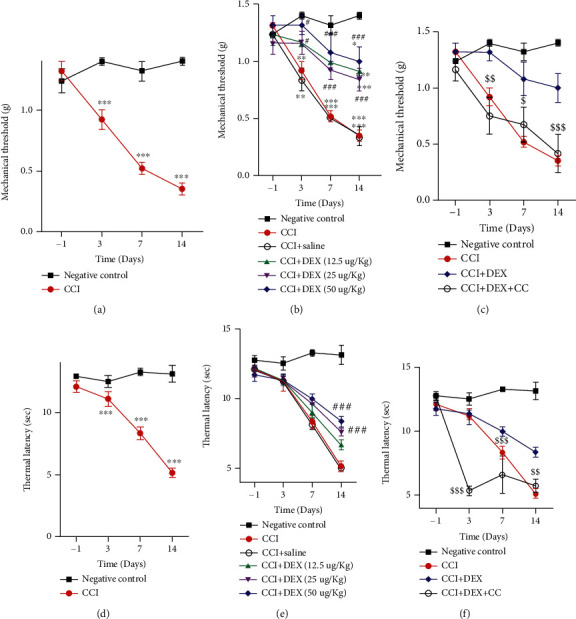
Impact of DEX on the pain thresholds among mice with CCI. (a) Gradually decreasing threshold for CCI-induced murine mechanical pain. (b) Effect of different doses of DEX on the mechanical pain threshold in CCI mice. (c) Effects of 25 *μ*g/kg DEX and AMPK inhibitor compound C on mechanical pain threshold in mice with CCI at various times. (d) CCI induced progressive decrease of thermal latency. (e) Effects of different doses of DEX on heat latency among mice with CCI. (f) Effects of 25 *μ*g/kg DEX and AMPK inhibitor compound C on the thermal latency in CCI mice at different times. Data are mean ± SD (*n* = 6); ^∗^*p* < 0.05, ^∗∗^*p* < 0.01, and ^∗∗∗^*p* < 0.001 versus negative control group; ^#^*p* < 0.05, ^##^*p* < 0.01, and ^###^*p* < 0.001 versus CCI group; ^$^*p* < 0.05, ^$$^*p* < 0.01, and ^$$$^*p* < 0.001 CCI+DEX versus CCI+DEX+CC group.

As observed in this study, DEX averted the decline of the CCI-elicited mechanical threshold, and the mechanical allodynia was mitigated for the CCI and CCI+saline groups in a time-responsive manner (Figure 1(a)). The reduction was particularly apparent on the 7^th^ and 14^th^ days (*p* < 0.001), in contrast to the negative control. The withdrawal threshold of the negative control varied insignificantly at arbitrary time points of inspection. Moreover, at the arbitrary time points of inspection, insignificant differences were noted between the CCI and CCI+saline groups.  Figure 1(b) shows that the CCI+DEX (25 *μ*g/kg) group had a higher PWMT starting on the 3^rd^ day than the CCI and CCI+saline groups (*p* < 0.05). From the 3^rd^ to 14^th^ day, DEX therapy significantly reduced the PWMT elevation in the CCI+DEX (25 *μ*g/kg) and CCI+DEX (50 *μ*g/kg) groups (*p* < 0.01, Figure 1(b)). Furthermore, the PWMT increased in the CCI+DEX (12.5 *μ*g/kg) group on the 14^th^ day (*p* < 0.01, Figure 1(b)), which was blocked by administering compound C (CC), an AMPK inhibitor, intraperitoneally at 10 mg/kg (*p* < 0.05, Figures 1(a) and 1(c)). However, DEX was unable to completely repress the PWMT alteration, as evidenced by the differences between the medication groups (CCI+DEX (12.5, 25, and 50 *μ*g/kg)) and the negative control (*p* < 0.001, Figure 1(b)), on the 14^th^ day.

Additionally, this study includes the measurement of PWTL. At various time points of surveillance (1^st^, 3^rd^, 7^th^, and 14^th^ day), the PWTLs were not considerably different for the negative control group (Figure 1(d)). For the CCI and CCI+saline groups, drastic declines in PWTLs were observed on the 3^rd^, 7^th^, and 14^th^ days following ligation in contrast to the negative control (*p* < 0.001, Figure 1(d)). The 3^rd^- and 7^th^-day values differed insignificantly among the CCI+saline, CCI, and CCI+DEX (12.5 *μ*g/kg) groups (*p* > 0.05, Figure 1(e)). Moreover, the 3^rd^-day postligation differences were insignificant among the CCI+saline, CCI, CCI+DEX (25 *μ*g/kg), and CCI+DEX (50 *μ*g/kg) groups (*p* > 0.05, Figure 1(e)). The elevation in the PWTL commenced on the 7^th^ day in the CCI+DEX (25 *μ*g/kg) and CCI+DEX (50 *μ*g/kg) groups (*p* < 0.01, Figure 1(e)), while the CC administration inhibited such elevation (*p* < 0.01, Figure 1(f)).

The variations in the thresholds for heat pain differed from those for mechanical pain, with prominently higher values in the CCI+DEX (25 and 50 *μ*g/kg) groups than those in the CCI+saline and CCI groups until 14 days (*p* < 0.05, Figure 1(e)). However, resumption to the level of the negative control seemed impossible (*p* < 0.05, Figure 1(e)), which was blocked by compound C (*p* < 0.01, Figure 1(f)). These results indicate that the resumption of the heat threshold is slower than that of the mechanical threshold. The favorable actions of DEX on the murine pain threshold were tightly associated with the therapeutic dosage and duration. However, at arbitrary time points of inspection, insignificant differences were noted between the 25 and 50 *μ*g/kg dose groups of CCI+DEX or between the CCI and CCI+saline groups. Based on these results, a 14-day time point and a DEX dose of 25 *μ*g/kg body weight were selected for the subsequent molecular and biochemical studies.

### 3.2. Perineural DEX Improved Mitochondrial Function and Exerted Antioxidative Potential in the Ischiadic Nerve of CCI Mice

**Figure 2:**
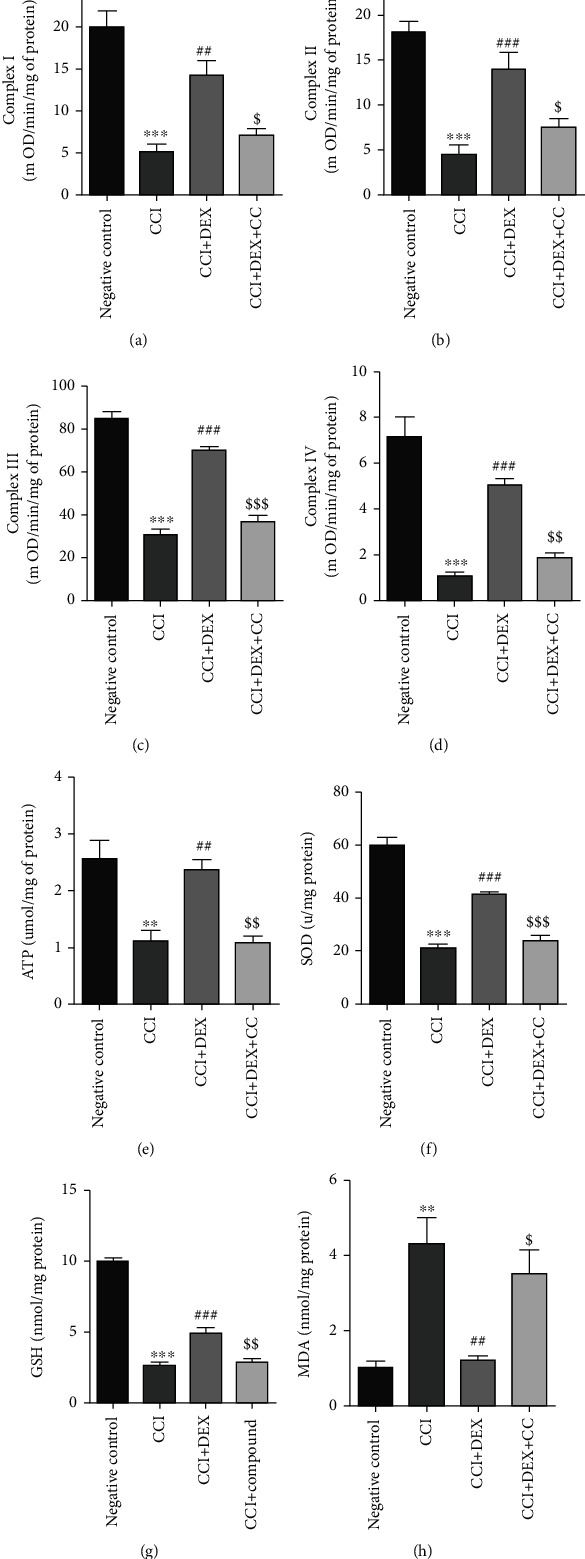
Impact of local injection of DEX around the sciatic nerve on oxidative phosphorylation and antioxidant stress ability of the mitochondria in the injured nerve of CCI mice. DEX increased the expression level of mitochondrial ETC complexes (a) I, (b) II, (c) III, and (d) IV in the injured nerve of CCI mice, and its effect could be partly inhibited by the AMPK inhibitor, compound C. DEX increased the (e) ATP and (f) SOD content in the injured nerve of CCI mice, and its effect was suppressive in part through the compound C (CC) injection. (g) DEX promoted GSH production in the injured nerve of CCI mice, and its effect was suppressive in part through the CC injection. (h) DEX inhibited GSH production in the injured nerve of CCI mice, and its effect was suppressive in part through the CC injection. Data presented are means ± SDs (*n* = 3) from triplicate separate experiments. ^∗∗^*p* < 0.01 and ^∗∗∗^*p* < 0.001 against negative control; ^##^*p* < 0.01 and ^###^*p* < 0.001 against CCI group; ^$^*p* < 0.05, ^$$^*p* < 0.01, and ^$$$^*p* < 0.001 CCI+DEX against CCI+DEX+CC groups.

It was observed that DEX significantly increased the complex I (*p* < 0.01, Figure 2(a)), II, III, and IV (*p* < 0.001, Figures 2(b)–2(d)) activities in the ischiadic nerves of the CCI mice. Furthermore, DEX significantly upregulated the level of ATP in the ischiadic nerve of CCI mice, indicating amelioration of the bioenergetic condition and mitochondrial functionality (*p* < 0.01, Figure 2(e)). The DEX treatment (*p* < 0.001, Figures 2(f) and 2(g)) restored the GSH and SOD levels, which had been significantly reduced in the CCI mice. The MDA levels were elevated prominently in the CCI mice and were restored by DEX (*p* < 0.01, Figure 2(h)). These effects of DEX were blocked by the administration of CC, an AMPK inhibitor (*p* < 0.001, Figures 2(c) and 2(f); *p* < 0.01, Figures 2(d), 2(e), and 2(g); *p* < 0.05, Figures 2(a), 2(b), and 2(h)).

### 3.3. DEX Upregulated the Ischiadic Nerve Expression of pAMPK in CCI Mice

**Figure 3:**
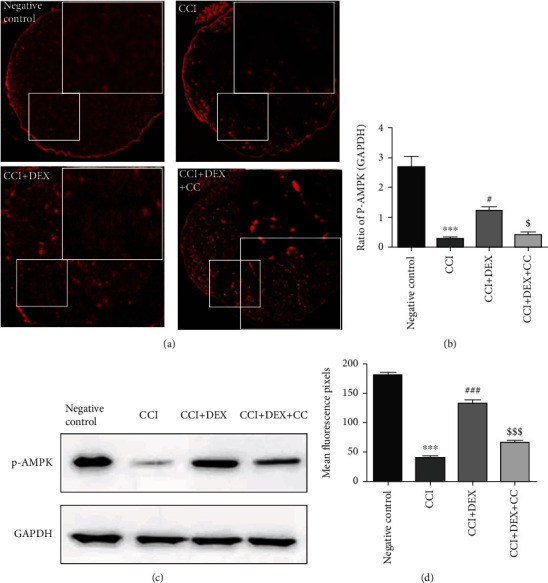
Impact of DEX on the pAMPK expression in the injured nerve of CCI mice. (a) Immunofluorescence localization showed that the local injection of DEX around the sciatic nerve elevated the pAMPK-positive cell counts prominently in the distal nerve after CCI. Micrographic magnification: ×100, scale bars: 50 *μ*m. (b) Histogram depicting an intergroup comparison of immunohistochemical scores. (c) Western blot was adopted to detect the expression of PAMPK in the distal nerve after CCI. (d) Densitometric quantification of pAMPK/GAPDH. Data are expressed as mean ± SD (*n* = 3) and represent three independent experiments. ^∗∗∗^*p* < 0.001 versus negative control group; ^#^*p* < 0.01 and ^###^*p* < 0.001 versus CCI group; ^$^*p* < 0.05 and ^$$$^*p* < 0.001 CCI+DEX versus CCI+DEX+CC group.

Both immunofluorescence assays and western blotting showed a significant reduction in the pAMPK level in the sciatic nerve of CCI mice (*p* < 0.001, Figures 3(a)–3(d)). It is observed that DEX significantly (*p* < 0.05, Figures 3(a) and 3(b); *p* < 0.001, Figures 3(c) and 3(d)) restored the levels when compared to those in the CCI-induced mice, which were suppressed in part through the CC administration (*p* < 0.05, Figures 3(a) and 3(b); *p* < 0.001, Figures 3(c) and 3(d)).

### 3.4. DEX Upregulated the Expression of OPA1 and SNPH but Not Drp1 or KIF5B in the Ischiadic Nerve of CCI Mice

**Figure 4:**
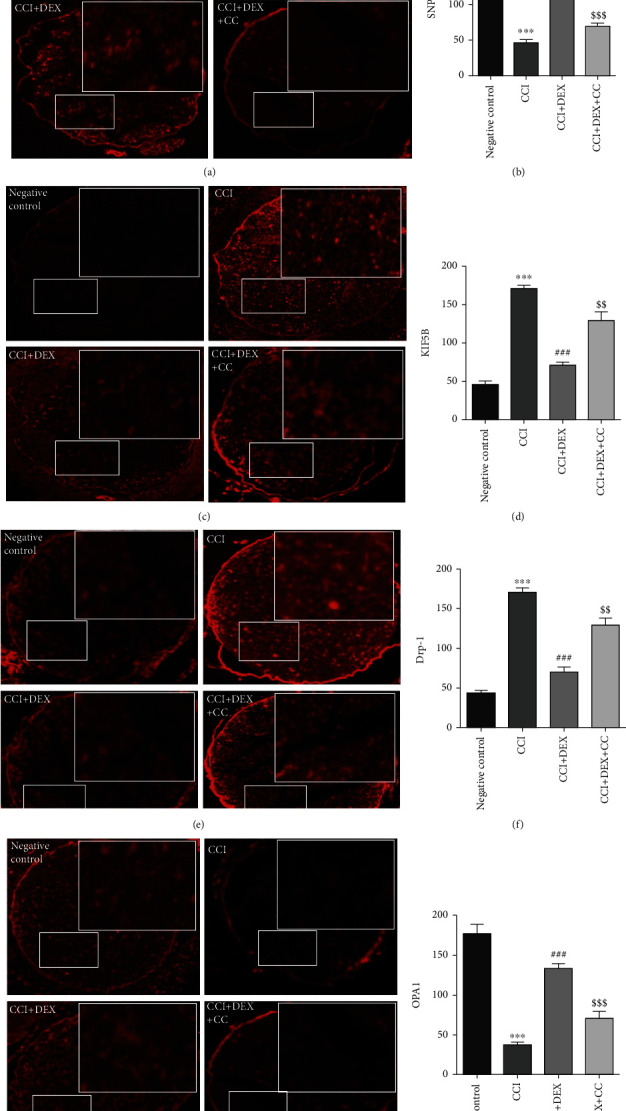
Immunofluorescence was used to detect the impact of local injection of DEX around the sciatic nerve on SNPH, KIF5B, Drp-1, and OPA1 expressions. (a) Immunofluorescence localization showed that local injection of DEX around the sciatic nerve significantly increased the number of SNPH and (g) OPA1 while lowering the number of (c) KIF5B- and (e) Drp-1-positive cells in the distal nerve after CCI. Photographs were taken at ×100 magnification. The scale presents a length of 50 *μ*m. The bar graph represents the immunohistochemical scores of various groups of (b) SNPH, (d) KIF5B, (f) Drp-1, and (h) OPA1. Data are mean ± SD (*n* = 3) and denote three independent experiments. ^∗∗∗^*p* < 0.001 versus negative control group; ^###^*p* < 0.001 versus CCI group; ^$$^*p* < 0.01 and ^$$$^*p* < 0.001 CCI+DEX versus CCI+DEX+CC group.

**Figure 5:**
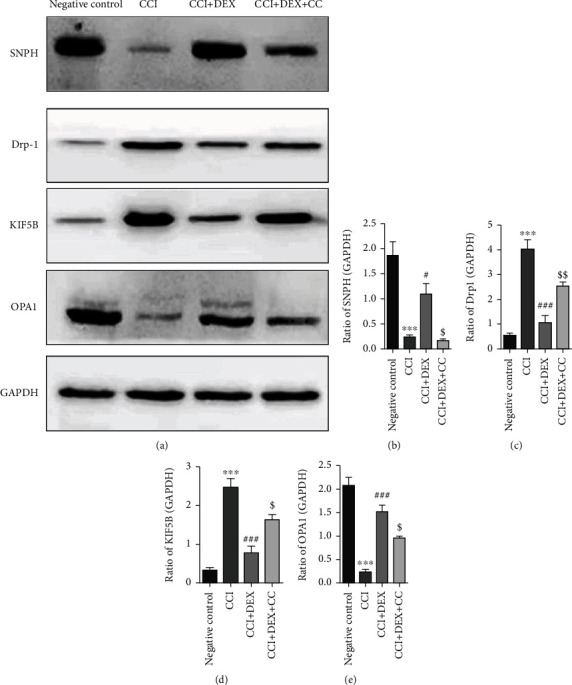
The impact of local injection of DEX around the sciatic nerve on the expressions of SNPH, KIF5B, Drp-1, and OPA1 was identified by the western blot analysis. (a) Western blot showed that the local injection of DEX around the sciatic nerve significantly enhanced the expression of SNPH and OPA1 and lowered the expression of KIF5B and Drp-1 in the distal nerve after CCI. Densitometric quantification of (b) SNPH/GAPDH, (c) KIF5B/GAPDH, (d) Drp-1/GAPDH, and (e) OPA1/GAPDH. Data are mean ± SD (*n* = 3) and represent three independent experiments. ^∗∗∗^*p* < 0.001 versus negative control group; ^#^*p* < 0.05 and ^###^*p* < 0.001 versus CCI group; ^$^*p* < 0.05 and ^$$^*p* < 0.01 CCI+DEX versus CCI+DEX+CC group.

In the sciatic nerve of CCI mice, both immunofluorescence assays and western blotting revealed a substantial decrease in the OPA1 and SNPH levels but an upregulation in the Drp1 and KIF5B levels (*p* < 0.001, Figures 4(a)–4(h), Figures 5(a)–5(e)). DEX significantly (*p* < 0.001, Figures 4(a)–4(h), Figures 5(a) and 5(c)–5(e); *p* < 0.05, Figures 5(a) and 5(b)) restored their levels in comparison to those in the CCI-induced mice, which were suppressed in part by CC administration (*p* < 0.001, Figures 4(a), 4(b), 4(g), and 4(h); *p* < 0.01, Figures 4(c)–4(f), Figures 5(a) and 5(c); *p* < 0.05, Figures 5(a), 5(b), 5(d), and 5(e)).

### 3.5. DEX Reduces Neuronal Activation and Inflammation in the DHSC of CCI Mice

**Figure 6:**
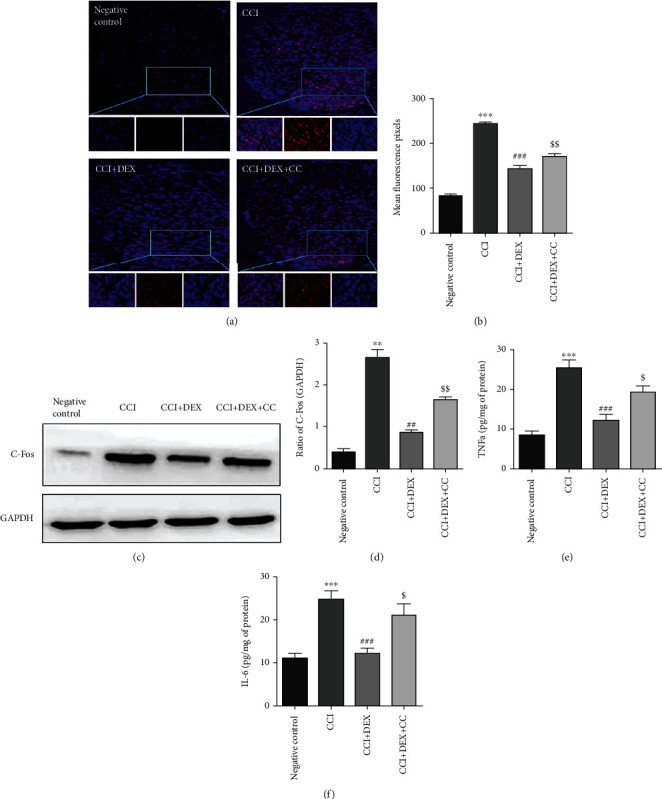
Effects of DEX on neuron-specific marker protein c-Fos and inflammatory mediators in the DHSC. (a) Immunofluorescence localization showed that local injection of DEX around the sciatic nerve significantly decreased the number of c-Fos-positive cells in the DHSC after CCI injuries. Photographs were taken at ×100 magnification. The scale shows a length of 50 *μ*m. (b) Bar graph represents immunohistochemical scores of various groups. (c) Western blot was used to detect the expression of c-Fos in the DHSC. (d) Densitometric quantification of c-Fos/GAPDH. Levels of (e) TNF-*α* and (f) IL-6 in the DHSC. Data are mean ± SD (*n* = 3) and represent three independent experiments. ^∗∗^*p* < 0.01 and ^∗∗∗^*p* < 0.001 versus negative control group; ^##^*p* < 0.01 and ^###^*p* < 0.001 versus CCI group; ^$^*p* < 0.05 and ^$$^*p* < 0.01 CCI+DEX versus CCI+DEX+CC group.

Both immunofluorescence assays and western blotting showed prominently elevated levels of c-Fos in the CCI mouse DHSC (*p* < 0.001, Figures 6(a) and 6(b); *p* < 0.01, Figures 6(c) and 6(d)). DEX significantly (*p* < 0.001, Figures 6(a) and 6(b); *p* < 0.01, Figures 6(c) and 6(d)) restored their levels when compared to the CCI-induced mice, which were suppressed in part by CC administration (*p* < 0.01, Figures 6(a)–6(d)).

TNF-*α* and IL-6 levels were found to be elevated in the DHSC of CCI mice (*p* < 0.001, Figures 6(e) and 6(f)) and were restored by DEX (*p* < 0.001, Figures 6(g) and 6(h)). The CC (*p* < 0.05, Figures 6(e) and 6(f)) prevented similar actions of DEX.

## 4. Discussion

In this paper, the effects of DEX administered to the perinerves in a CCI-induced neuropathic pain mouse model were explored. The findings revealed that ① DEX attenuated the mechanical threshold and the heat latency of pain induced by the CCI operation. ② DEX inhibited neuronal excitation and inflammatory cytokine secretion in the spinal dorsal cord through upregulation of the AMPK pathway. ③ The effects of DEX on CCI mice are mediated by the AMPK-mediated restoration of mitochondrial function, which includes inhibition of abnormal mitochondrial transport and improved mitochondrial fission in situ. Even though this was only an animal experiment with small sample sizes in vivo, it indicated that DEX can be utilized in the perinerves to treat CCI-elicited neuropathic pain, and its safety and efficacy may be better than systemic DEX in future clinical trials.

Over a 14-day course, progressive decline in the mechanical pain threshold and heat pain latency were monitored in the ipsilateral posterior limbs of the CCI mice. Such declines were averted by the DEX therapy, confirming findings from prior studies that employed intraperitoneally injected DEX [12–15]. The local injection of DEX around the sciatic nerve can reduce the dosage and systemic side effects, such as somnolence and hypertension. Furthermore, it can increase the duration of the tissue action, especially in combination with local anesthetics. In the current experiment, significant enhancements in the PWMT and PWTL were observed, especially on the 14^th^ day. The heat sensitivity threshold was reestablished at a slower rate than the mechanical pain threshold. The results demonstrate that early DEX administration to the impaired nerves can enhance the murine pain threshold.

Mitochondrial disorder influences proinflammatory signaling and vice versa [14, 16]. Disorderly mitochondria, which are capable of intensifying oxidative stress, can facilitate a malicious cycle of inflammatory impairment. Inflammatory cascades, which are mediators of proinflammatory cytokine secretion, can disrupt the ETC protein activity and lower ATP generation [16, 17]. According to our study, in addition to preventing ETC disorder and enhancing ATP generation, the perinerve DEX enhanced mitochondrial antioxidation by increasing the levels of MDA, SOD, and GSH in the ischiadic nerve in mice with CCI-induced neuropathic pain.

AMPK, as a heterotrimeric Ser/Thr kinase, is stimulated by variations in the cellular ratio of AMP to ATP, which acts as an energy sensor to regulate oxidative stress and energy homeostasis, promoting mitochondrial functionality and repressing degenerative nerve diseases [17, 18]. The anti-inflammatory effect of AMPK activation has been revealed, which was achieved by lowering the levels of neuron-mediated inflammatory cytokines [14]. The repressive role of AMPK on neuropathic pain development has been demonstrated in various animal experiments [19–22]. In this paper, DEX therapy significantly sustained the sciatic nerve levels of AMPK phosphorylation in mice with CCI-induced neuropathic pain. As implied by the aforementioned research outcomes, DEX has neuronal deactivating and mitoprotective properties, which can help prevent behavioral and functional deficiencies. According to current findings, DEX could stimulate AMPK directly.

A question arises as to whether AMPK is necessary for repressing the CCI-elicited neuropathic pain mediated by DEX. Therefore, we investigated whether the action of DEX was reversible by CC. The findings revealed that mechanical allodynia which was mitigated due to DEX was significantly reversed by CC on the 7^th^ day (Figure 1(b)). Furthermore, the thermal hyperalgesia mitigated by DEX was prominently reversed by CC on the 14^th^ day after CCI (Figure 1(d)). Therefore, the key mechanism by which DEX produces antinociceptive effects in pain hypersensitivity states is AMPK activation. Recent reports support our assertion of the pain-influencing role of AMPK in animal models of neuropathy and acute nociception [19, 23, 24]. However, the specific mechanism by which AMPK activation in ischiadic nerves preserves mitochondrial function, exerts antioxidative stress, and combats inflammation remains elusive.

The critical role of AMPK in regulating the dynamic mitochondrial equilibrium has been recently demonstrated [25, 26]. Mitochondrial dynamics refers to the fusion and fission kinetics of mitochondria, the mechanisms by which mitochondria self-renew and self-repair in response to damage and aging. Meanwhile, mitochondrial axonal transport (MAT) is a key approach to supplementing an insufficient number and functional decline of local mitochondria [27]. The majority of the mitochondria in normal cells are anchored at fixed positions. When local damage or inflammation causes a high local energy demand, a significant number of surrounding mitochondria are detached from their original positions and transported to the damaged site to replenish the lack of local mitochondria [28].

The effects of MAT on cells are both beneficial and detrimental. MAT can provide energy to repair the damaged site, and it can also cause a lack of energy supply in other parts of the cell or even the whole cell. Consequently, we hypothesize that activating AMPK can promote in situ division and fusion of damaged mitochondria, restore the local energy supply, and reduce abnormal MAT, thus protecting mitochondrial function and antioxidative stress.

In this paper, the expression of the anchoring proteins, syntaphilin (SNPH) and kinesin-KIF5B, which are the major regulatory proteins of MAT in the sciatic nerve, was explored. Using the immunohistochemical and western blot methods, it was found that DEX downregulated the expression of KIF5B and upregulated the expression of SNPH in the CCI murine sciatic nerve. According to a report, SNPH played a pivotal role in the AMPK pathway-dependent mediation of mitochondrial anchorage, and the expression levels of AMPK and SNPH were positively correlated [29, 30].

SNPH promotes activity-dependent axonal mitochondrial fixation by binding to KIF5 [31], while AMPK phosphorylates KIF5 and inhibits mitochondrial forward transport [32]. The decrease in KIF5 expression results in a decrease in mitochondrial forward transport capacity. However, the expression of the axon mitochondrial anchor-protein SNPH, which acts as a brake in mitochondria, will increase [31]. Caino et al. reported that decreased SNPH in a carcinoma model leads to increased oxidative stress, decreased cellular multiplication, and enhanced cellular locomotion [33]. Our findings reveal that the activation of AMPK can reduce sciatic nerve MAT by downregulating KIF5 and upregulating SNPH, enabling in situ mitochondrial reactivation and antioxidative stress. The foregoing studies confirm our hypothesis and the experimental results.

Furthermore, the expressions of the major regulatory proteins of mitochondrial dynamics, OPA1 (fusion protein) and Drp1 (fission protein), in the affected sciatic nerve were examined. The results indicated that DEX downregulated Drp1 and upregulated OPA1 expression in the sciatic nerve of CCI mice. According to Jae et al., SNPH ubiquitination suppressed the migration of mitochondria and the fusion-fission cycles for organelles by anchoring SNPH on tubulin.

Moreover, when SNPH ubiquitination is hindered, the mitochondrial dynamics are stimulated, resulting in the enhanced mitochondrial Drp1 recruitment and the Drp1-dependent migration of carcinoma cells [34]. An essential factor in the trafficking of mitochondria is active Drp1 [35]. Dale et al. discovered increased DRG neuronal expressions of mitochondrial fission proteins in mice with a high-fat diet- (HFD-) triggered painful diabetic neuropathy (PDN) [36]. These results are consistent with our findings. In diabetic rats, activation of neurons in DHSC is found to have a significant impact on the inflammatory reactions to NPP [37]. The suppressed TNF-*α*, IL-1*β*, and IL-6 secretions in DHSC contributed to the curative potential of DEX [15]. These cytokines, which are capable of directly sensitizing nociceptors, have a significant impact on hyperalgesia and allodynia, such as pain perception [38, 39].

Currently, the main objective of NPP therapy is symptom mitigation, which is achieved through hyperexcitable neuronal repression. However, targeting disorderly mitochondria and neurocyte inflammation offers a plethora of therapeutic options since it abolishes actual neuropathologies that facilitate chronic pain development. As the first relay station, DHSC is responsible for transferring the pain information to the center, which is crucial in the formation of pathological neuralgia. The expression of c-Fos in DHSCs is associated with the nociceptive response and is considered a neuronal marker of pain. In the CCI neuropathic pain mouse model, perinerve DEX significantly inhibited the levels of c-Fos in DHSCs. Moreover, the TNF-*α*, IL-1*β*, and IL-6 secretions were weakened by DEX, suggesting its probable suppressive effects on the neuron-mediated inflammation and oxidative stress in DHSC. A prior report on neurogenic pain attenuation by inhibiting the release of inflammatory substances from neurons in DHSC corroborates our findings [37].

## 5. Conclusion

In summary, this paper describes the impact of perinerve DEX on CCI-induced neuropathic pain and its underlying molecular mechanisms. Furthermore, the administration of DEX can alleviate the CCI-elicited NPP in mice by the activation of AMPK, which inhibits the activation of DHSC neurons and benefits from restoring the function of mitochondria in the peripheral nerve and attenuating inflammation in DHSC.

## Data Availability

All data could be found in the manuscript and previous literatures in the reference.
